# Emergency Management of Pelvic Bleeding

**DOI:** 10.3390/jcm10010129

**Published:** 2021-01-01

**Authors:** Simone Frassini, Shailvi Gupta, Stefano Granieri, Stefania Cimbanassi, Fabrizio Sammartano, Thomas M. Scalea, Osvaldo Chiara

**Affiliations:** 1 General Surgery and Trauma Team, ASST Grande Ospedale Metropolitano Niguarda, University of Milan, Milano, Piazza Ospedale Maggiore 3, 20162 Milan, Italy; simo.frassi@gmail.com (S.F.); steff.granieri@gmail.com (S.G.); scimbanassi@yahoo.it (S.C.); fabriziosammartano@hotmail.it (F.S.); 2 Adams Cowley Shock Trauma Center, University of Maryland, 22 S Greene St, Baltimore, MD 21201, USA; shailvi.gupta@som.umaryland.edu (S.G.); tscalea@som.umaryland.edu (T.M.S.)

**Keywords:** pelvic trauma, packing, extra-peritoneal packing, REBOA, resuscitation, bleeding

## Abstract

Pelvic trauma continues to have a high mortality rate despite damage control techniques for bleeding control. The aim of our study was to evaluate how Extra-peritoneal Pelvic Packing (EPP) and Resuscitative Endovascular Balloon Occlusion of the Aorta (REBOA) impact the efficacy on mortality and hemodynamic impact. We retrospectively evaluated patients who sustained blunt trauma, pelvic fracture and hemodynamic instability from 2002 to 2018. We excluded a concomitant severe brain injury, resuscitative thoracotomy, penetrating trauma and age below 14 years old. The study population was divided in EPP and REBOA Zone III group. Propensity score matching was used to adjust baseline differences and then a one-to-one matched analysis was performed. We selected 83 patients, 10 for group: survival rate was higher in EPP group, but not significantly in each outcome we analyzed (24 h, 7 day, overall). EPP had a significant increase in main arterial pressure after procedure (+20.13 mmHg, *p* < 0.001), but this was not as great as the improvement seen in the REBOA group (+45.10 mmHg, *p* < 0.001). EPP and REBOA are effective and improve hemodynamic status: both are reasonable first steps in a multidisciplinary management. Zone I REBOA may be useful in patients ‘in extremis condition’ with multiple sites of torso hemorrhage, particularly those in extremis.

## 1. Introduction

Management of traumatic pelvic fractures is one of the most complex challenges for trauma surgeons. The mortality rate remains high in hemodynamically unstable patients after an acute post-traumatic pelvic hemorrhage. The mortality rate can be greater than 40% due to rapid exsanguination [1,2]. 

A multimodal treatment approach in pelvic trauma has been the gold standard. This includes an early mechanical stabilization with pelvic binder, when necessary, and then both operative management—Extra-Peritoneal Packing (EPP)—and endovascular interventions—such as Angio-Embolization procedures (AE) or Resuscitative Endovascular Balloon Occlusion of the Aorta (REBOA), placed zone III. Every type of emergency treatment must be considered in according with the stability, force direction and patoanathomy of pelvic fractures, looking for example to ‘Tile’ or ‘Young and Burgess’ classification. [3,4,5,6,7]. Many management guidelines and scientific reports have been published proposing alternative treatment algorithms including all these interventions; the cornerstone of all these algorithms is the hemodynamic status of the patient.

Angiography and subsequent embolization controls anywhere from 80% to 100% of arterial hemorrhage related to pelvic trauma, in addition to mechanical stabilization. Despite this, however, arterial bleeding occurs only in 15–25% of cases [8,9].

EPP has been identified recently as an effective and fast surgical procedure to control bleeding in hemodynamically unstable pelvic features [10,11]. EPP was first described in 1994 in Germany. More recent evidence supports extraperitoneal pelvic as a life-saving procedure reducing mortality compared with patients managed with other damage control techniques. One of the key-concepts of EPP is the control of major venous bleeding by direct compression; as venous bleeding makes up about 75–80% of pelvic hemorrhage [12]. EPP can be performed in less than 20 min, safely either in the OR or in the Emergency Department (ED).

Recently, REBOA has emerged as a promising technique for bleeding control in patients in hemorrhagic shock. When the balloon is deployed in Zone 3, it was proposed as an alternative to control pelvic hemorrhage [13,14]. Some studies have demonstrated a survival benefit with REBOA specifically when used in Zone 3 as opposed to Zone 1 for an effective control of pelvic arterial flow [15]. However, the balloon is not universally available and data from other studies suggest REBOA is associated with severe complication such as ischemia-reperfusion syndrome, acute kidney injury, amputation and increased mortality [16].

At present, there are several guidelines for the emergency management of pelvic bleeding after severe trauma injury, from a lot of different associations and societies. The aim of our study was to compare the efficacy and outcome of damage control techniques, following a previous study from our group about EPP and extending our analysis to REBOA [17]. Our primary endpoint was to evaluate the mortality rate with EPP and REBOA, the secondary endpoint is the hemodynamic effect of the procedures.

## 2. Materials and Methods

### 2.1. Study Design and Setting

This study was a retrospective analysis of Trauma Registries at two institutions, ASST Grande Ospedale Metropolitano Niguarda, University of Milan and R Adams Cowley Shock Trauma Center, University of Maryland, in Baltimore. The collection of data and the work was conducted at the ASST Niguarda Trauma Center, University of Milan, in accordance with local ethical committee, from 2002 to 2018.

### 2.2. Study Population

For patient selection, we followed the inclusion criteria of the previous work from our group, presented at the 78th Annual Meeting of American Association for the Surgery of Trauma in September 2019 in Dallas, Texas [17].

All trauma patients sustaining blunt trauma with a pelvic fracture and hemodynamic instability from 2002 to 2018 were included. For the second part of the study, comparing EPP to REBOA, we considered only Zone III REBOA patients. We evaluated demographic data, mechanism of trauma, Systolic Blood Pressure (SBP), Mean Arterial Pressure (MAP), Injury Severity Score (ISS), need for damage control laparotomy, type of pelvic fracture, associated extra-pelvic injuries, head, chest, abdomen and extremities Abbreviated Injury Scale (AIS, 2005 version) score, EPP related infections, length of stay in Intensive Care Unit (ICU), REBOA place Zone, REBOA complications, length of hospitalization (LOS), 24 h and overall mortality.

**Figure 1:**
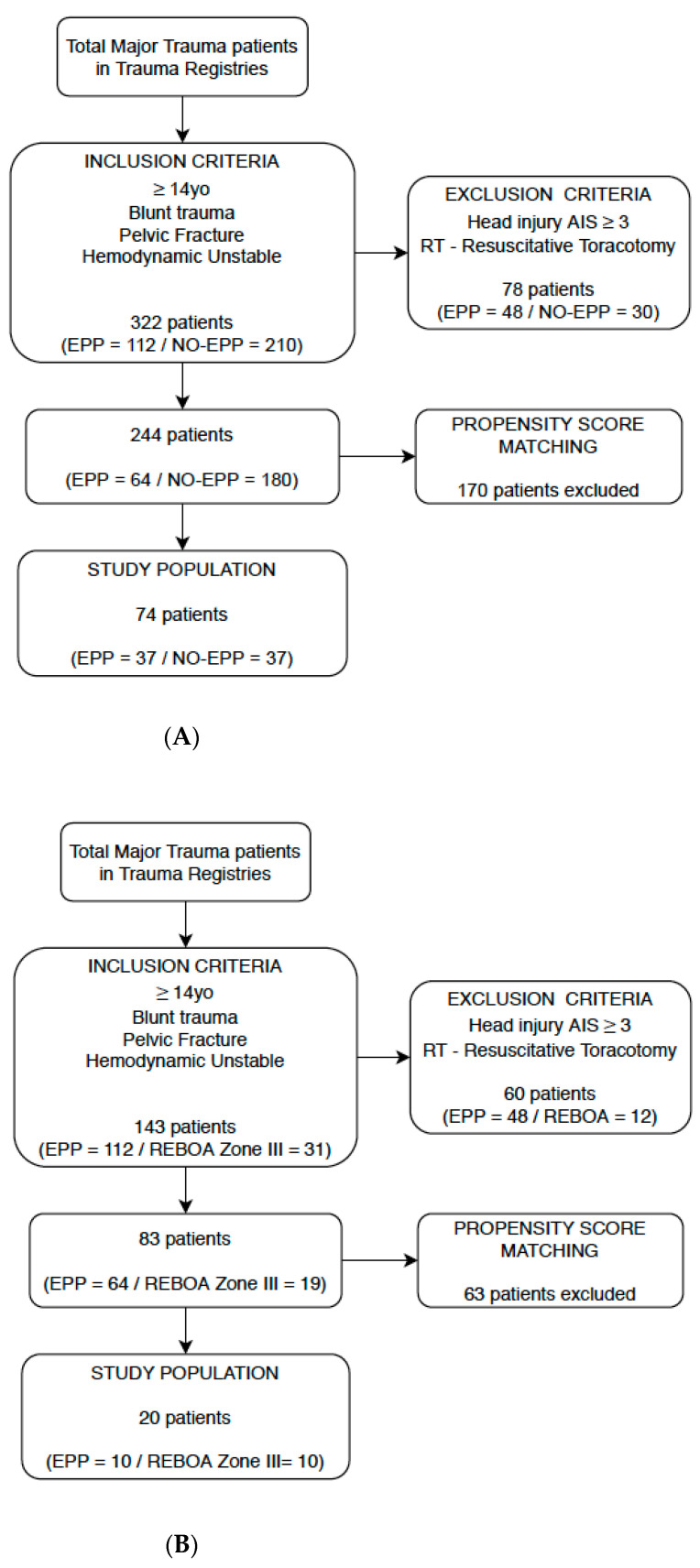
(**A**) Flow Diagram of population selection in EPP vs. NO-EPP section. (**B**) Flow Diagram of population selection in EPP vs. REBOA section.

Hemodynamic instability was defined as a systolic blood pressure <90 mmHg despite pelvic binder, adequate fluid resuscitation (1000 mL of intravenous crystalloids as stated by Advanced Trauma Life Support—ATLS manual) and transfusion of ≥2 units of Packed Red Blood Cells (PRBCs) [18]. The pattern of pelvic fracture was classified according to Tile and Young & Burgess classifications. Exclusion criteria were a concomitant severe brain injury (AIS brain > 3), patients who underwent resuscitative thoracotomy, penetrating trauma and age below 14 years (Figure 1A,B).

### 2.3. Treatment Protocol

We identified in our study three different treatment protocols according to the period and the Institution we were looking at.

**Figure 2:**
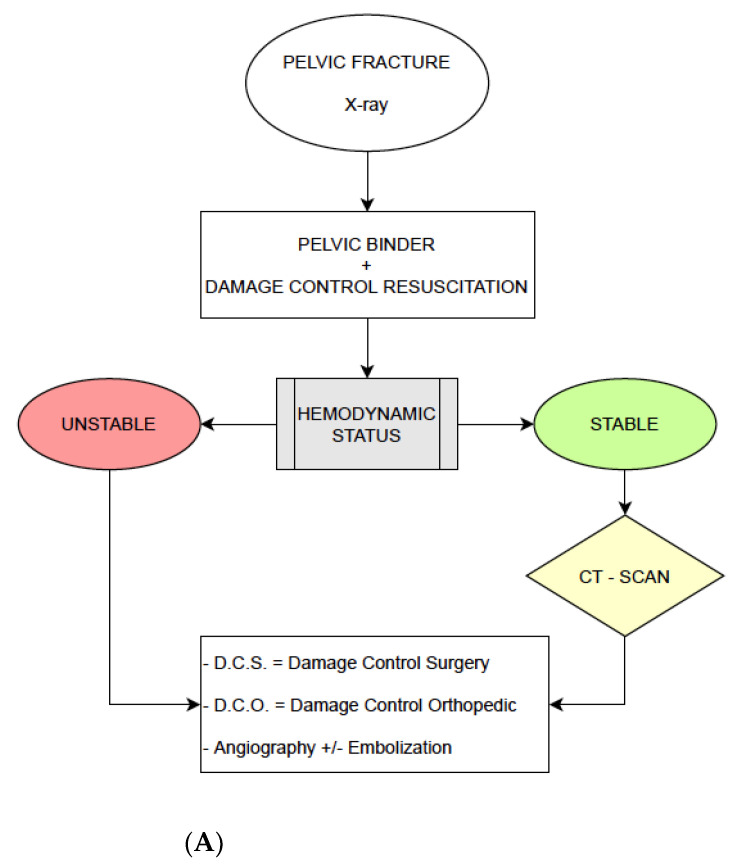

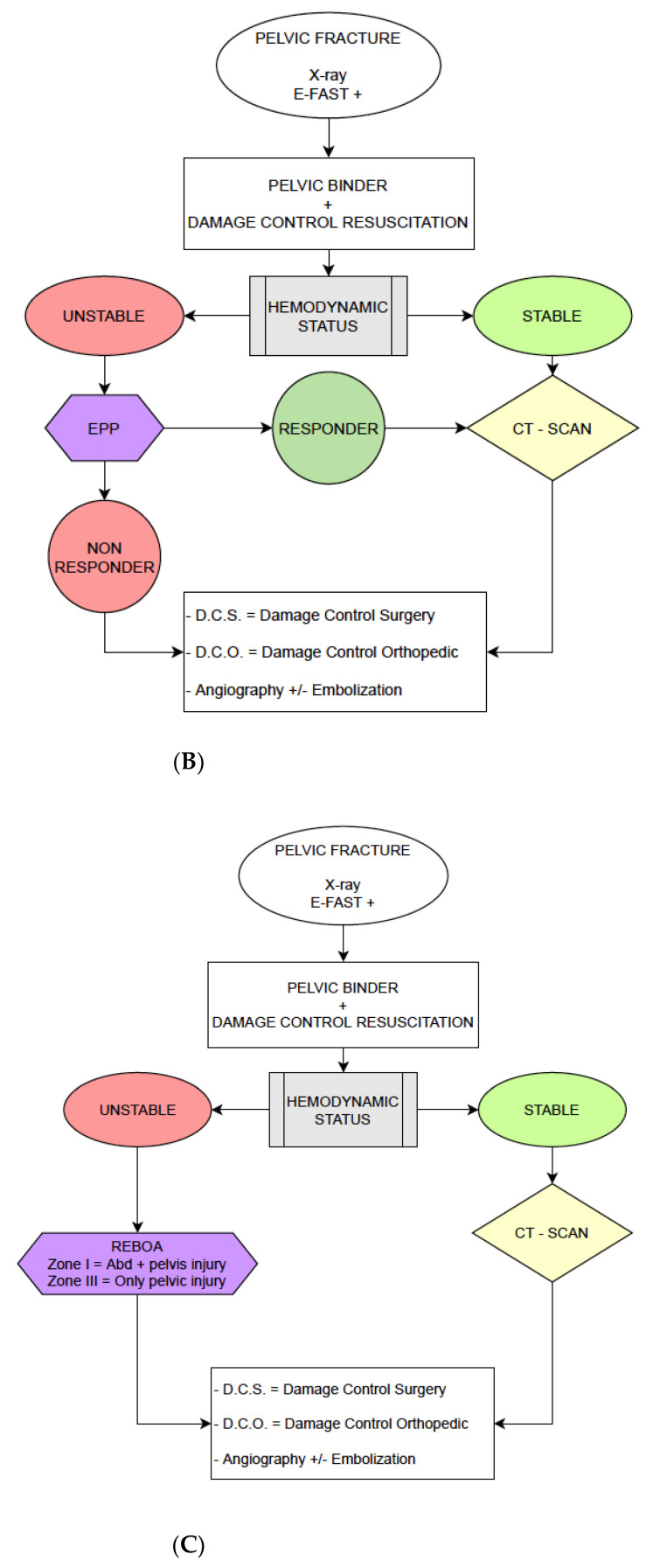
(**A**) Treatment protocol of No-EPP group, until 2009. (**B**) Treatment protocol of EPP group, 2009–2018. (**C**) Treatment protocol of REBOA group, 2014–2018.

Firstly, we considered the management of patients at the ASST Niguarda Trauma Center until 2009, following the model our group explained in the previous work we cited. In patients with pelvic fractures and a hemodynamically unstable condition, a pelvic binder was placed compressing at the level of great trochanters and a Damage Control Resuscitation with fluids and blood products was started. If Extended-Focus Assessment with Sonography for Trauma (E-FAST) exam was positive for free pelvic fluid and X-ray confirmed a pelvic fracture, confirming an unstable condition, we used to access directly to the OR to perform laparotomy, AE and external fixation of the fracture if necessary (Figure 2A).

In 2009, early EPP was introduced in the Niguarda treatment protocol. In patients with a positive E-FAST and an X-RAY confirming a pelvic fracture, if after placement of a pelvic binder and the immediate transfusion of at least two units of 0-negative PRBCs was still hypotensive, this patient had immediate EPP. After the procedure, if the patient’s hemodynamics improved, a contrast-enhanced computed tomography (CT-Scan) was performed. Angiography was performed when CT-Scan demonstrated contrast extravasation and, if confirmed as arterial, embolization was performed. If the patient did not respond to EPP and remained unstable, it was mandatory directly to access to the OR for surgical, endovascular and/or orthopedic procedures, as appropriate (Figure 2B).

Extra-Peritoneal Packing was even performed in ED/Trauma Bay if the patient was too unstable to be safely transported to OR.

In the second part of the study, we collaborated with R Adams Cowley Trauma Center in Baltimore, evaluating the REBOA protocol they used from 2013 to 2018 in patients with pelvic injury with unstable hemodynamic condition. If the patient was hypotensive, E-FAST positive and—if performed—an X-ray with pelvic fracture, REBOA was placed in Zone I in case of suspected abdominal-pelvic injury or Zone III in case of a suspected isolated pelvic injury. After placement and balloon insufflation, imaging could be performed or not before the access to the OR, as dictated by the patients’ hemodynamics. In our series, we considered only patients with Zone III REBOA or patients with Zone I REBOA that was immediately re-positioned in to Zone III (Figure 2C).

### 2.4. Statistical Analysis

Following the design in the previous work from our group, we considered the statistical design already published and the sample in the first part of the analysis was split into a no-EPP and an EPP group. The second part of the work we carried forward had a similar design and the same statistical analysis: we split the patients into an EPP and REBOA group.

Data were recorded in a computerized spreadsheet (Microsoft Excel 2016; Microsoft Corporation, Redmond, WA, USA) and analyzed with statistical software (IBM Corp. Released 2017. IBM SPSS Statistics for Windows, Version 25.0. Armonk, NY, USA). Propensity Score Matching (PSM) was performed to adjust for differences in the baseline characteristics in the two groups [19,20]. A one-to-one nearest neighbor logistic regression matching model was built setting the maximum tolerated difference between matched subjects (caliper) at 0.1 standard deviation (SD). Age, ISS, damage control laparotomy, abdominal and extremities injuries AIS score were selected as potential confounders and entered in the model. Graphical (histogram of propensity score, dot plot of standardized differences) and mathematical (standardized differences) balance diagnostics were evaluated after matching for an accurate assessment of the goodness of our model.

The distribution of the sample per each variable of interest was assessed with the Shapiro–Wilk test. Differences in proportions were evaluated with Pearson’s χ^2^ or Fisher’s test, whereas independent samples Mann–Whitney test and Wilcoxon signed-rank test for repeated measurements were used to compare continuous variables.

A *p*-value < 0.05 was considered statistically significant.

## 3. Results

A total of 8374 major trauma patients were admitted to Niguarda Trauma Center in the period of our study. In the previous work from our group, we analyzed 322 patients according to our inclusion criteria: after exclusion criteria and Propensity Score Matching our study population was seventy-four patients, 37 in no-EPP and 37 in EPP group. We demonstrated Extra-Peritoneal Packing was an effective procedure, improving 24 h and overall survival in contrast with no-EPP group (*p* = 0.042, *p* = 0.047). We also evidenced the time from ED admission to the hemostatic procedure was significantly shorter in the EPP group (49.43 min, *p* < 0.001) and hemodynamic improvement before and after packing was performed. The comparison between measurements of pre- and post- procedural SBP and MAP (respectively 65.87 ± 21.5 vs. 94.25 ± 32.54 mmHg and 49.92 ± 17.12 vs. 70.05 ± 25.07 mmHg) demonstrates a highly significant increase in hemodynamic condition (*p* < 0.001) (Table 1).

In the second part of this work, we considered the EPP group in Niguarda Trauma Center—64 patients comparing them to the 19 patients treated with REBOA group at the R Adams Cowley Shock Trauma Center. The most frequent mechanism of trauma was fall (36.14%) and fifty-five patients in the study were male (66.27%); patients in EPP group had more severe injuries with a mean ISS of 42.85 ± 10 versus 30.94 ± 10.52 in the REBOA group (*p* = 0.022).

**Figure 3:**
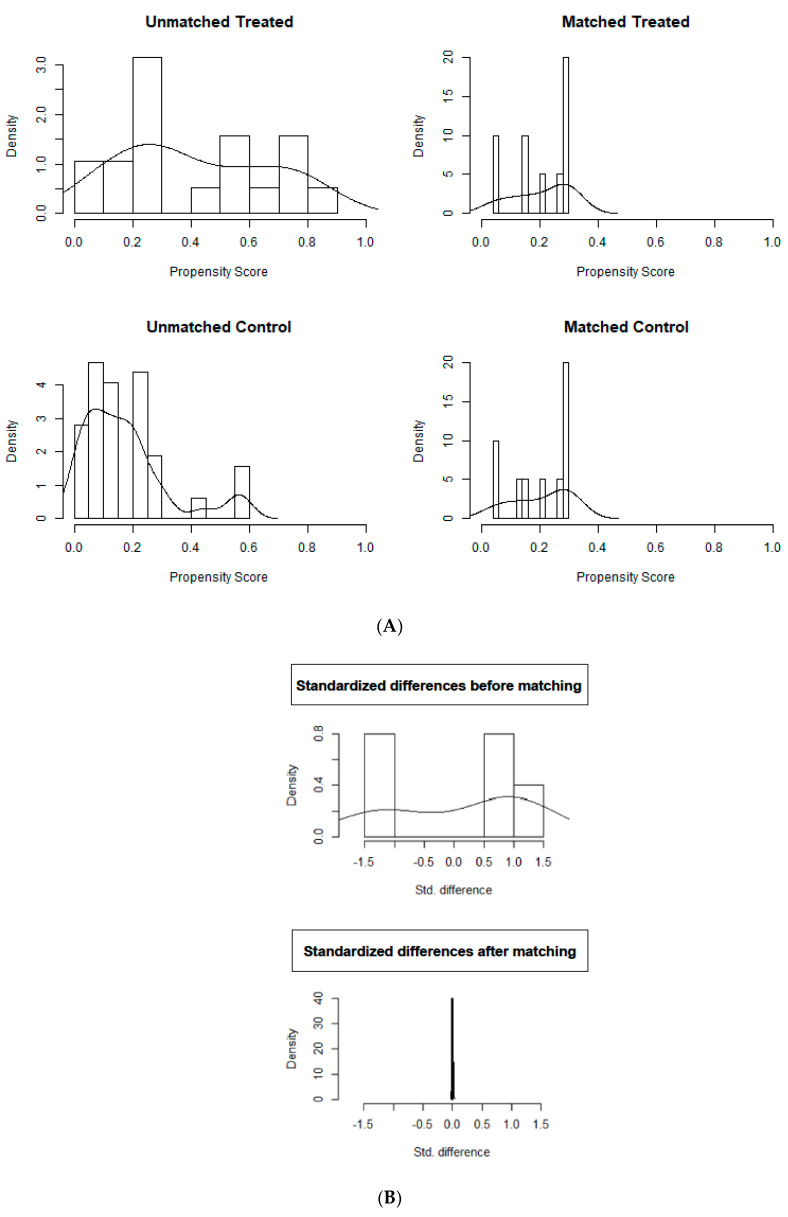
(**A**) Histograms of propensity score. (**B**) Histograms of propensity score.

The EPP group and REBOA group were balanced according to a PSM with nineteen possible couples of patients before matching: for a more objective evaluation we computed standardized differences of selective confounders and for all covariates we observed a small effect size, defined by a standardized difference value below 0.2 after matching. A total of 10 couples was eligible at the end of the matching, and graphical assessment of balance before and after matching is displayed in Figure 3A,B.

Six patients out of twenty died in the first 24 h: 2/10 in the EPP group (20.0%) compared to 4/10 (40.0%) in REBOA group (*p* = 0.337). After seven days of hospitalization, we registered one more patient died in the REBOA group (2/10 versus 5/10, *p* = 0.171); overall mortality rate was 30.0% (3/10) in the EPP group, compared to 60.0% (6/10) in the REBOA group. While survival rate with Extra-Peritoneal Pelvic Packing this did not reach statistical significance (*p* = 0.185) (Table 2).

For our secondary outcome, we considered the whole EPP subset (64 patients) and all the REBOA group included in the study (19 patients): we evaluated the hemodynamic improvement before and after the procedure was performed. As reported in Table 3, we subdivided analysis between total, survived and dead patients for each group. Patients surviving after EPP procedure had an increase in MAP of 24.69 mmHg compared to patients not surviving with an increase of 11.95 mmHg: total EPP group evidenced a hemodynamic improvement of 20.13 mmHg, from a pre-procedural value of 49.92 ± 17.12 mmHg to a post-procedural value of 70.05 ± 25.07 mmHg (*p* < 0.001).

In the REBOA group, surviving patients registered a hemodynamic improvement in MAP of 36.40 mmHg (58.30 ± 12.32 mmHg pre-procedural versus 94.70 ± 12.59 mmHg post-procedural; *p* < 0.001) compared to an increase of 54.77 mmHg in non-surviving patients (31.11 ± 33.82 mmHg vs. 85.88 ± 57.52 mmHg; *p* = 0.014). Considering the whole REBOA group, 19 patients, pre-procedural MAP of 45.42 ± 27.90 mmHg improved to a post-procedural value of 90.52 ± 39.45 mmHg demonstrating a highly significant increase in blood pressure of 45.10 mmHg (*p* < 0.001) (Table 3).

## 4. Discussion

Bleeding control in severe pelvic trauma has been one of the most controversial topics in the recent trauma and emergency surgery literature.

In some trauma centers, the approach is still a mechanical stabilization of the fracture and—if necessary—endovascular control of the bleeding via angio-embolization. While AE has the advantage of providing definitive hemostasis, it takes time to assemble the resources necessary and perform the procedure. EPP and REBOA have emerged as damage control techniques to achieve hemodynamic improvement in critically injured patients in order to ‘gain time’ for definitive hemostatic procedures.

Our study takes origin from the largest EPP series in Europe, comparing firstly this technique to a previous protocol without damage control approach and then, in the second part of the study, we compared packing to a small series of patients treated with REBOA placed in zone III. There are some studies comparing REBOA with other damage control techniques—in particular EPP—on larger size like Trauma Quality Improvement Program in USA [21]. On the other hand, our current study completes the work of our group and analyzes every emergency maneuver on hemodynamically unstable pelvic trauma from 2002 to 2018.

Currently, WSES, EAST and the American College of Surgeons consider both pelvic packing and REBOA for severe pelvic trauma management, and they can be used also in combination, before definitive endovascular hemostatic procedure and mechanical stabilization [1,2]. The surgical technique suggested for EPP is a vertical or transverse incision with a consequent vertical division of the fascia; after the pelvic hematoma is evacuated, laparotomy pads are placed on each side of the bladder adjacent to the pelvic ring to perform a direct compression of the bleeding site. Another recent work from our group demonstrated EPP can be even performed in Emergency Department instead of OR, without an increased risk of an infection complication [22]. REBOA is considered a damage control technique for patients with severe torso hemorrhage, and it can be placed zone I—from left subclavian to celiac trunk—or zone III—from the lowest renal artery to aortic bifurcation—in case of isolated pelvic bleeding [21]. Some studies evidenced a survival benefit when the balloon is placed zone III, but there is also a clear association with late mortality and major complications like renal injuries, vascular damages and limb amputations with the use of REBOA [23,24,25].

The comparison between EPP and No-EPP was already published in our previous work [17].

In the current study, when comparing the EPP-group and REBOA-group, we observed differences in early and overall mortality. In the first 24 h, we observed a survival rate of 80.0% in the group treated with packing compared to a 60.0% in the REBOA group. The overall survival rate was 70.0% in EPP-group and 40.0% with REBOA. However, these differences did not reach significant significance. Thus, we can make no statement about any mortality advantage or disadvantage with either technique. Both seemed to improve hemodynamics.

A recent paper by Mikdad S. et al. reviewed a 3-year patient series of the Trauma Quality Improvement Program (TQIP) in the USA, comparing the efficacy of packing and REBOA in pelvic trauma patients to control bleeding: they used a statistical design similar to our—Propensity Score Matching. In that study, 103 patients were studied and the authors observed a significant in-hospital mortality higher in patients receiving REBOA (52.0% vs. 37.3%; *p* = 0.048) [21].

Our secondary endpoint was the hemodynamic impact of the procedure, looking at the MAP after EPP and REBOA. In our series we registered a highly significant improvement in main arterial pressure before and after packing (+20.13 mmHg; *p* < 0.001) but it was even higher in patients with REBOA (+45.10 mmHg; *p* < 0.001).

To our knowledge, there are other papers reporting data about hemodynamic impact of REBOA in resuscitation [26], but our work is the first observing efficacy of both technique (EPP and REBOA) on main arterial pressure and comparing them specifically.

This study has several weaknesses. Firstly, it is a retrospective analysis of data. Secondly, our population was not large enough to obtain significant results on our primary outcome: other studies are needed to clarify the relative roles of REBOA and EPP. Finally, our Italian center—at the time we are writing—is just beginning to begin to use REBOA. It is too early in that experience to utilize it for the REBOA arm. Thus, we performed multi-center study.

Thus, it would seem that both EPP and REBOA are effective in the early treatment of patients with pelvic fracture bleeding and hypotension. The institutions involved in this study are both high volume centers but there are a number of differences, as well. Not all of these can be controlled for in any single study. The use of either technique requires institutional commitment and surgeon training. While each can be learned relatively quickly, attempting to perform either for the first time in the middle of the night seems unwise. Most importantly, neither technique likely is the best for all patients. It would seem that EPP, if done quickly would suffice for patients with largely venous bleeding. It seems unlikely to be the best choice for patients with major arterial bleeding. REBOA, on the other hand does not stop bleeding in most cases. It does achieve temporary hemodynamic stability, particularly in those with arterial bleeding while the patient is resuscitated and plans are made for other means of hemostasis, as needed. Most importantly, both of these techniques cannot solve this complex problem in isolation. Both must be part of a well-coordinated, multi-disciplinary effort in order to be effective.

**Figure 4:**
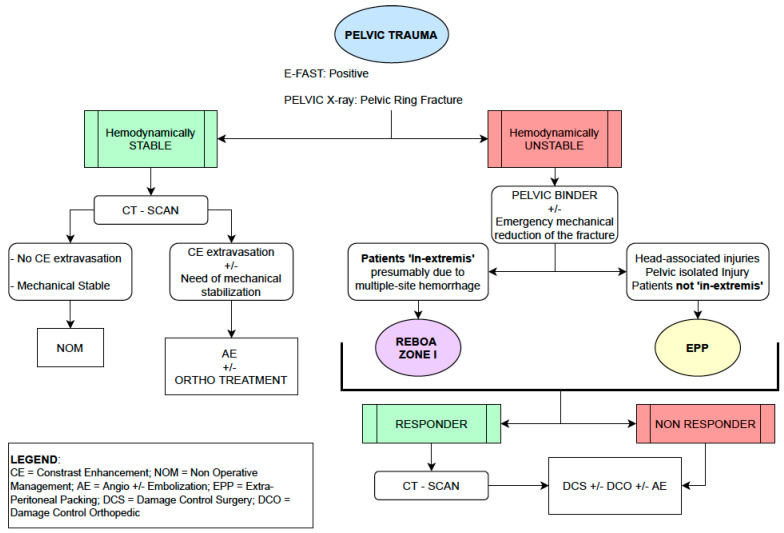
Pelvic Trauma Management Algorithm.

Our idea—considering a setting in which devices and experience are available—is that a multi-disciplinary management should be considered gold standard with complementary surgical and endovascular approach: in case of hemodynamic unstable trauma with isolated pelvic or head-associated injuries, EPP should be standard of care. When patients ‘in extremis’ due to torso hemorrhage, REBOA—placed zone I—should be preferred. In cases of multiple sites trauma, a 360° approach with availability and application of both techniques should be ideal. Our group coordinator, corresponding author of this work, was involved in Italian National Institute of Health guidelines and we summarized his recommendations in a flowchart attached below (Figure 4) [27].

## 5. Conclusions

Extra-peritoneal pelvic packing and REBOA are both effective procedures that improve hemodynamic status in pelvic unstable severe injury. There is no clear survival advantage to either technique. The choice of hemostatic maneuvers should be made based on institutional resources and local practice. Additional study will be necessary in order to define any real advantages to either technique.

## Figures and Tables

**Table 1:** Comparisons between No-EPP and EPP groups after PS Matching.

Variables	No-EPP (*n* = 37)		EPP (*n* = 37)		*p*
	Number/Mean	%/SD	Number/Mean	%/SD	
Gender (male)	27	73	22	59.5	0.32
Mechanism of Trauma					<0.001 *
Road accident	27	73	9	24.3	
Fall	9	24.3	15	40.6	
Pedestrian	0	0	9	24.3	
Cyclist	0	0	4	10.8	
Domestic accident	1	2.7	0	0	
ICU-LOS (days)	12	17.25	30.35	35.57	0.02 *
Length of hospitalization	33.65	34.97	64	55.79	0.012 *
N. of angioembolizations	21	56.8	21	56.8	1
N. of external fixations	21	56.8	27	73	0.22
Time to hemostatic procedure (mins) **	155.9	95.11	49.43	24.97	<0.001 *
SBP in Emergency Department	82.95	27.9	65.73	17.79	0.004 *
HR in Emergency Department	97.32	30.53	105.03	22.83	0.33
N. of PRBCs < 24 h	11.28	10.18	8.08	6.74	0.36
RTS	6.06	1.69	5.28	1.98	0.07
Probability of death (TRISS)	50.69	31.75	57.02	31.12	0.33
Deaths ≤ 24 h	15	40.54	7	18.92	0.042 *
Total deaths	16	43.24	8	21.62	0.047 *

* Significant value; ** Time elapsed from the admission in ED to the end of the hemostatic procedure. ICU-LOS: Length of Stay in Intensive Care Unit; SBP: Systolic Blood Pressure; HR: Heart Rate; PRBCs: Packed Red Blood Cells; RTS: Revised Trauma Score; TRISS: Trauma and Injury Severity Score.

**Table 2:** Primary outcome EPP vs. REBOA group at 24 h, 7 days, overall.

	EPP		REBOA		*p* Value	OR (Odds Ratio)	CI (Confidence Interval) 95%
	*Survived*	*Dead*	*Survived*	*Dead*			
**OUTCOME 24 h**	8 80.00%	2 20.00%	6 60.00%	4 40.00%	0.337	2.66	0.361–19.712
**OUTCOME 7 days**	8 80.00%	2 20.00%	5 50.00%	5 50.00%	0.171	4.00	0.550–29.096
**OUTCOME OVERALL**	7 70.00%	3 30.00%	4 40.00%	6 60.00%	0.185	3.50	0.549–22.304

**Table 3:** Hemodynamic impact of procedure.

	*n* Patients	MAP PRE-Procedure (mmHg)	MAP POST-Procedure (mmHg)	Δ (mmHg)	*p* Value
EPP-GROUP total	64	49.92 ± 17.12	70.05 ± 25.07	+20.13	<0.001
EPP-GROUP Survivied	42/64	54.71 ± 22.30	79.40 ± 20.04	+24.69	<0.001
EPP-GROUP Died	22/64	40.41 ± 14.54	52.36 ± 14.88	+11.95	0.06
REBOA-GROUP Total	19	45.42 ± 27.90	90.52 ± 39.54	+45.10	<0.001
REBOA-GROUP Survived	10/19	58.30 ± 12.32	94.70 ± 12.59	+36.40	<0.001
REBOA-GROUP Died	9/19	31.11 ± 33.82	85.88 ± 57.52	+54.77	0.014

## Data Availability

The data presented in this study are available on request to the corresponding author.

## Reporting Checklist

The authors present the study in accordance with the STROBE reporting checklist [28].

## References

[1] Coccolini F., Stahel P.F., Montori G., Biffl W., Hörer T., Catena F., Kluger Y., Moore E.E., Peitzman A.B., Ivatury R.R. (2017). Pelvic trauma: WSES classification and guidelines. World J. Emerg. Surg..

[2] Cullinane D.C., Schiller H.J., Zielinski M.D., Bilaniuk J.W., Collier B.R., Como J., Holevar M., Sabater E.A., Andrew Sems S., Matthew Vassy W. (2011). Eastern Association for the Surgery of Trauma practice management guidelines for hemorrhage in pelvic fracture—update and systematic review. J. Trauma Acute Care Surg..

[3] Tran T.L., Brasel K.J., Karmy-Jones R., Rowell S., Schreiber M.A., Shatz D.V., Albrecht R.M., Cohen M.J., DeMoya M.A., Biffl W.L. (2016). Western Trauma Association critical decision in trauma: Management of pelvic fracture with hemodynamic instability—2016 updates. J. Trauma Acute Care Surg..

[4] Costantini T.W., Coimbra R., Holcomb J.B., Podbielski J.M., Catalano R.D., Blackburn A., Scalea T.M., Stein D.M., Williams L., Conflitti J. (2017). Pelvic fracture pattern predicts the need for hemorrhage control intervention—results of an AAST multi-institutional study. J. Trauma Acute Care Surg..

[5] Tosounidis T.I., Giannoudis P.V. (2013). Pelvic fractures presenting with hemodynamic instability: Treatment options and outcomes. Surgeon.

[6] Croce M.A., Magnotti L.J., Savage S., Wood G.W., Fabian T.C. (2007). Emergent Pelvic Fixation in Patient with Exsanguinating Pelvic Fractures. J. Am. Coll. Surg..

[7] Scaglione M., Parchi P.D., DiGrandi G., Latessa M., Guido G. (2010). External fixation in pelvic fractures. Musculoskelet. Surg..

[8] Tesoriero R.B., Bruns B.R. (2017). Angiographic embolization for hemorrhage following pelvic fracture: Is it “time” for a paradigm shift?. J. Trauma Acute Care Surg..

[9] Osborn P.M., Smith W.R., Moore E.E., Cothren C.C., Morgan S., Williams N.A.E., Stahel P.F. (2009). Direct retroperitoneal pelvic packing versus pelvic angiography: A comparison of two management protocols for haemodynamically unstable pelvic fractures. Injury.

[10] Cothren C.C., Osborn P.M., Moore E.E., Morgan S.J., Johnson J.L., Smith W.R. (2007). Preperitoneal pelvic packing for hemodynamically unstable pelvic fractures: A paradigm shift. J. Trauma Acute Care Surg..

[11] Chiara O., Di Fratta E., Mariani A., Michaela B., Prestini L., Sammartano F., Cimbanassi S. (2016). Efficacy of extra-peritoneal pelvic packing in hemodynamically unstable pelvic fractures, a propensity score analysis. World J. Emerg. Surg..

[12] Burlew C.C., Moore E.E., Stahel P.F., Geddes A.E., Wagenaar A.E., Pieracci F.M., Fox C.J., Campion E.M., Johnson J.L., Mauffrey C. (2017). Preperitoneal pelvic packing reduces mortality in patients with life-threatening hemorrhage due to unstable pelvic fractures. J. Trauma Acute Care Surg..

[13] Brenner M., Teeter W., Hoehn M., Pasley J., Hu P., Yang S., Romagnoli A., Diaz J., Stein D., Scalea T. (2018). Use of resuscitative endovascular balloon occlusion of the aorta for proximal aortic control in patients with severe hemorrhage and arrest. JAMA Surg..

[14] Morrison J.J., Percival T.J., Markov N.P., Villamaria C., Scott D.J., Saches K.A., Spencer J.R., Rasmussen T.E. (2012). Aortic balloon occlusion is effective in controlling pelvic hemorrhage. J. Surg. Res..

[15] Do W.S., Forte D.M., Sheldon R.R., Weiss J.B., Barron M.R., Sokol K.K., Black G.E., Hegge S.R., Eckert M.J., Martin M.J. (2019). Preperitoneal balloon tamponade and resuscitative endovascular balloon occlusion of the aorta: Alternatives to open packing for pelvic-associated hemorrhage. J. Trauma Acute Care Surg..

[16] DuBose J.J., Scalea T.M., Brenner M., Skiada D., Inaba K., Cannon J., Moore L., Holcomb J., Turay D., Arbabi C.N. (2016). The AAST prospective Aortic Occlusion for Resuscitation in Trauma and Acute Care Surgery (AORTA) registry: Data on contemporary utilization and outcomes of aortic occlusion and resuscitative balloon occlusion of the aorta (REBOA). J. Trauma Acute Care Surg..

[17] Frassini S.S., Gupta S.S., Granieri S.S., Cimbanassi S.S., Sammartano F.F., Scalea T.M., O Chiara O. (2020). Extraperitoneal packing in unstable blunt pelvic trauma: A single-center study. J. Trauma Acute Care Surg..

[18] Ramenofsky M., Bell R. (2018). ATLS: Advanced Trauma Life Support.

[19] Rosenbaum P.R., Rubin D.B. (1983). The central role of the propensity score in observational studies for casual effect. Biometrika.

[20] Pearl J. (2009). Understanding propensity scores. Casuality: Models, Reasoning and Inference.

[21] Mikdad S., Inge van Erpe A.M., Moheb M.E., Fawley J., Saillant N., King D.R., Kaafarani H.M.A., Velmahos G., Mendoza A.E. (2020). Pre-Peritoneal Pelvic Packing for Early Hemorrhage Control Reduces Mortality Compared to Resuscitative Endovascular Balloon Occlusion of the Aorta in Severe Blunt Pelvic Trauma Patients: A National-wide Analysis. Injury.

[22] Reitano E., Granieri S., Frassini S., Sammartano F., Cimbanassi S., Chiara O. (2020). Infectious complications of extra-peritoneal pelvic packing in emergency room. Updates Surg..

[23] Biffl W.L., Fox C.J., Moore E. (2015). The role of REBOA in the control of exsanguinating torso hemorrhage. J. Trauma Acute Care Surg..

[24] Yamamoto R., Cestero R.F., Suzuki M., Funabiki T., Sasaki J. (2019). Resuscitative endovascular balloon occlusion of the aorta (REBOA) is associated with improved survival in severely injured patients: A propensity score matching analysis. Am. J. Surg..

[25] Brenner M., Inaba K., Aiolfi A., DuBose J., Fabian T., Bee T., Holcomb J.B., Moore L., Skarupa D., Scalea T.M. (2018). Resuscitative Endovascular Balloon Occlusion of the Aorta and Resuscitative Thoracotomy in select patients with hemorrhagic shock: Early results from the American Association for the Surgery of Trauma Aortic Occlusion in Resuscitation for Trauma and Acute Care Surgery Registry. J. Am. Coll. Surg..

[26] Madurska M., Ross J.D., Scalea T.M., Morrison J.J. (2020). State-of-the-Art Review—Endovascular Resuscitation. Shock.

[27] ISS (2020). Linea Guida sulla Gestione Integrata del Trauma Maggiore Dalla Scena dell’evento alla Cura Definitive. https://snlg.iss.it.

[28] von Elm E., Altman D.G., Egger M., Pocock S.J., Gøtzsche P.C., Vandenbroucke J.P., STROBE Initiative (2007). The Strengthening the Reporting of Observational Studies in Epidemiology (STROBE) Statements: Guidelines for Reporting Observational Studies. PLOS Med..

